# Testing the relationships between facial emotion recognition, levels of Autistic traits, and eye gaze patterns

**DOI:** 10.3389/fnhum.2026.1824976

**Published:** 2026-08-24

**Authors:** Jayden Z. Li, Fan Yang, Marilyn Chege, Victoria Moir, Fakhri Shafai, Julia Montenegro, Naomi Dodgson, Leila Moradkhani, Meara Stow, Tse Wing Winnie Ho, Arin E. Abraham, Elizabeth Gateman, Samantha E. Schulz, Ryan A. Stevenson

**Affiliations:** 1 Faculty of Science, University of Western Ontario, London, ON, Canada; 2 Western Centre for Brain and Mind, University of Western Ontario, London, ON, Canada; 3 Western Institute for Neuroscience, University of Western Ontario, London, ON, Canada; 4 Department of Neuroscience, University of Western Ontario, London, ON, Canada; 5 Department of Psychology, University of Western Ontario, London, ON, Canada; 6 Faculty of Education, University of Western Ontario, London, ON, Canada; 7 Centre for Vision Research, York University, North York, ON, Canada

**Keywords:** autism, emotion, affective, multisensory, eye tracking

## Abstract

Individuals with high levels of Autistic traits often experience challenges in interpreting others’ emotions, potentially stemming from atypical patterns of visual attention to facial cues. This study investigates whether levels of Autistic traits in a non-clinical sample are associated with overall emotion recognition accuracy, differential attention to facial areas of interest (eyes, mouth, and other regions), and emotion-specific recognition impairments. Eighty-one participants were presented with videos of actors displaying the six basic emotions (anger, disgust, fear, happiness, sadness, surprise) at high and low intensities while eye movements were recorded. Levels of Autistic traits were quantified using a composite score derived from well-validated questionnaires. Overall, higher levels of Autistic traits correlated with weaker emotion recognition performance (*r* = −0.30, *p* = 0.006). Attention to the mouth was positively correlated with emotion recognition accuracy (*r* = 0.27, *p* = 0.014), but not to the eyes. Notably, levels of Autistic traits did not correlate with gaze patterns to either mouth or eye regions, a null finding that differs from established literature on eye avoidance patterns in Autistic populations. Levels of Autistic traits were selectively associated with recognition of negative emotions of anger, disgust, and fear, but not happiness. These findings confirm the relationship between Autistic traits and emotion recognition difficulties in the broader population, as well as the importance of attention to emotionally salient facial features in emotion recognition. However, our hypothesis that eye-gaze patterns would mediate the relationship between autistic traits and emotion recognition difficulties was not supported.

## 1 Introduction

Effective social interaction relies on the ability to accurately interpret others’ emotions (Keltner et al., 2022; Liu et al., 2023; Cowen et al., 2021). These emotions are conveyed through a rich tapestry of affective cues, including facial expressions, vocal tone, and subtle nonverbal signals that reveal a speaker’s intentions, feelings, and expectations (Scherer, 2003; Liu et al., 2023; Sauter et al., 2010). An individual’s ability to decode these signals leads to improved social communication, and reduced emotion recognition may lead to social isolation and exclusion (De la Torre-Luque et al., 2022; Takemoto et al., 2025; Geng et al., 2025; Team, 2025; Morrison et al., 2019; Van Kleef and Cote, 2021).

Difficulties with facial emotion recognition have been reported in many neurodevelopmental populations, including Autism Spectrum Disorder (ASD; Ji et al., 2025; Schnitzler et al., 2024; Dollion et al., 2022; Ghanouni and Eves, 2025; Uljarevic and Hamilton, 2013). Although decreased emotion recognition is not a formal diagnostic criterion for ASD (American Psychiatric Association, 2022; Uljarević et al., 2022), facial emotion recognition in general is reportedly weakened in individuals with ASD (Ji et al., 2025; Schnitzler et al., 2024; Dollion et al., 2022; Jelili et al., 2021). There is also evidence suggesting that Autistic individuals have difficulties identifying certain emotions more than others. For example, recognition accuracy of happiness is often comparable to their non-Autistic peers, whereas more difficulties are often reported with anger, fear, and disgust (Ji et al., 2025; Schnitzler et al., 2024; Dollion et al., 2022; Jelili et al., 2021). Approximately 1 in 36 children in North America are diagnosed with ASD, highlighting the societal importance of addressing these difficulties (Shaw, 2025). Consequently, improving the understanding of these difficulties holds significant clinical and educational relevance, as insights will lead to more informed social skills supports and potential training programs.

Unlike the many clinical investigations in ASD, how Autistic traits in the general population relate to emotion recognition is not extensively studied. A handful of studies have found similar results in neurotypical adults with a high degree of Autistic traits, in which individuals with higher levels of Autistic traits have shown similar difficulties with emotion recognition (Wang et al., 2024; Lewis et al., 2024; Keating et al., 2022; Macinska et al., 2024; Carter Leno et al., 2023). This suggests that the relationship between Autism and emotion recognition may extend to those with high Autistic traits among the broader population (Mihailov et al., 2020; Qiao et al., 2025; Lin et al., 2020; Folatti et al., 2024; Buehler et al., 2024). Despite the clinical relevance, the subthreshold population remains less extensively studied compared to diagnosed ASD investigations (Qiao et al., 2025; Mihailov et al., 2020; Wang et al., 2024; Carter Leno et al., 2023).

One prominent theory suggests that the emotion recognition difficulties observed in Autistic individuals stem from atypical patterns of visual attention to facial cues (Black et al., 2017; Maes et al., 2024; Reisinger et al., 2020). Specifically, Autistic individuals tend to allocate less visual attention to the eyes and compensate by focusing more on the mouth region (Brien, 2015; Maes et al., 2024; Stuart et al., 2023; Kim et al., 2022; Wang et al., 2024; Buehler et al., 2024). Notably, accurate emotion recognition is also associated with distinct gaze patterns: for example, negative emotions such as anger and fear are more effectively identified by information from the eyes, while positive emotions like happiness are more closely associated with mouth cues (Kim et al., 2022; Šķilters et al., 2024; Chen et al., 2015; Calvo and Nummenmaa, 2016). This means that autistic individuals’ preference for the mouth over the eyes may leave them at a disadvantage for recognizing negative emotions but allows for preserved recognition of happiness relative to non-Autistic individuals (Ji et al., 2025; Jelili et al., 2021; Kim et al., 2022; Schnitzler et al., 2024). To our knowledge, these gaze patterns and their impact on emotion recognition accuracy have not been thoroughly examined in subclinical populations. Since individuals with high Autistic traits exhibit difficulties with emotion recognition similar to those diagnosed with Autism (Wang et al., 2024; Lewis et al., 2024; Keating et al., 2022; Macinska et al., 2024; Carter Leno et al., 2023), the visual attention theory may also explain the emotion recognition difficulties in individuals with high levels of Autistic traits in the broader population.

In the present study, we aim to study how visual attention to different facial regions affects the recognition of distinct facial emotion expressions and how this visual attention pattern is related to autistic traits in a broader population. Our primary goals are to test (1) whether emotion recognition is related to levels of Autistic traits in the general public, (2) whether gaze distribution to the eyes and/or mouth is related to emotion recognition, and (3) if goals 1 and 2 are significant, to test whether patterns of eye gaze mediate the relationship between levels of Autistic traits and emotion recognition. Secondarily, where significant results are found in our primary goals, we will follow up with examinations of whether these relationships are specific to specific emotions.

## 2 Methods

### 2.1 Participants

A total of 81 participants were included in the final analysis, with an additional 19 recruited but excluded from analysis (see below for details). Participants were recruited from the undergraduate psychology participant pool at Western University. The sample ranged in age from 17 to 46 years (M = 19.05, SD = 3.48), with 76% identifying as female and 23% as male. Notably, all but one participant fell within the range of 17–21 years old. Regarding handedness, 87% of participants were right-handed and 6% were left-handed. The majority of participants (96%) reported English as their first language. All participants reported having normal or corrected-to-normal visual acuity and typical hearing abilities. The study procedures received approval from Western University’s Research Ethics Board and adhered to the ethical principles outlined in the 2013 Declaration of Helsinki.

### 2.2 Apparatus

Data collection was conducted using E-Prime 3.0 software. Eye movements were recorded via a Tobii Pro X3-120 Hz screen-mounted eye-tracker positioned beneath the display monitor. Participants maintained a fixed viewing distance of 49 cm using a chin rest stabilization system. Audio presentation occurred through Logitech R-10S-0152A1 external speakers calibrated to deliver stimuli at an average of approximately 52.9 decibels.

**Figure 1:**
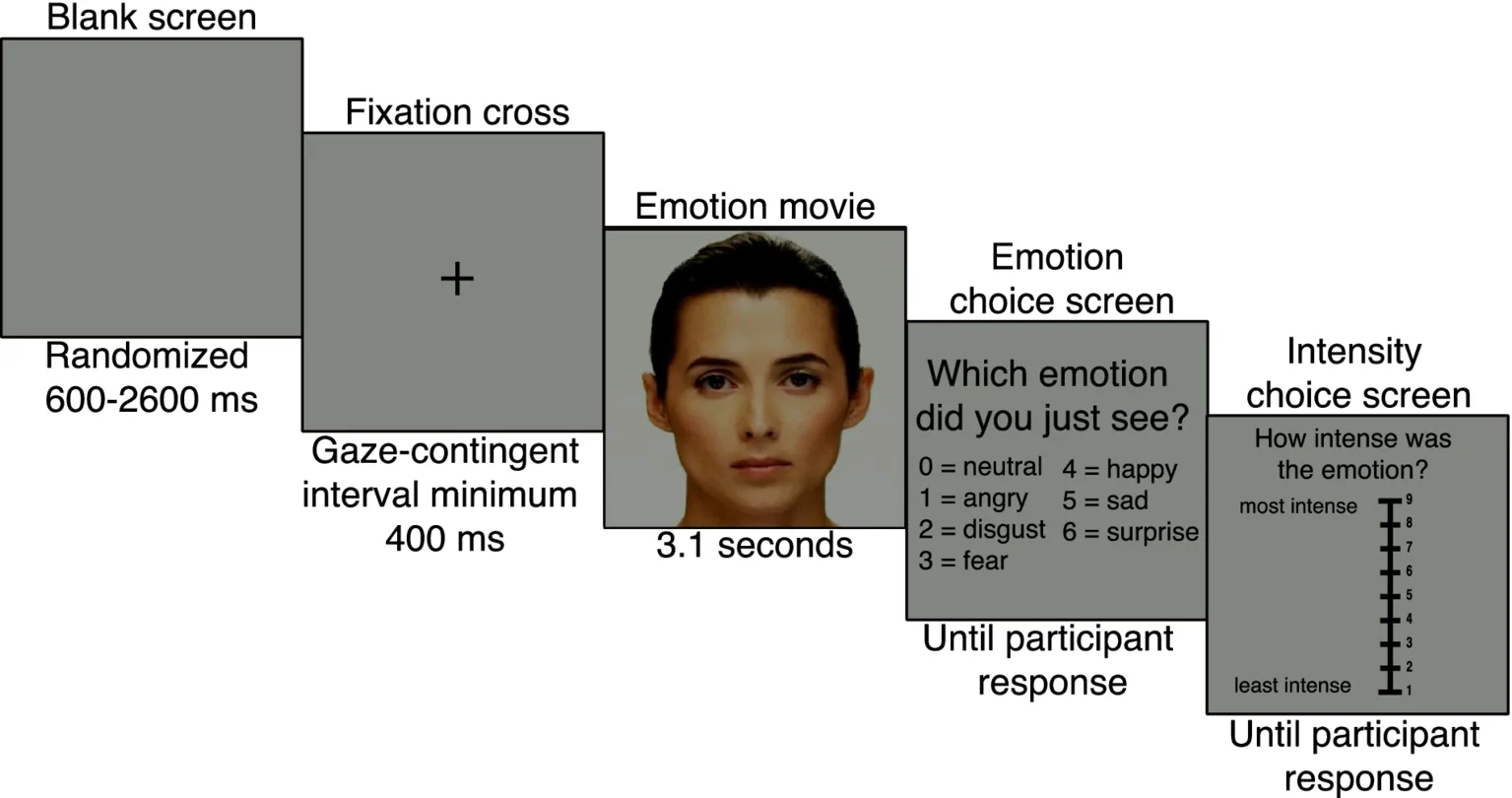
Each trial began with a blank screen (randomized 600–2,600 ms), followed by a gaze-contingent fixation cross (minimum 400 ms) and a 3.1-s emotion video. Participants then selected the emotion category and rated emotional intensity; both response screens remained onscreen until a response was made. Stimulus image reproduced from Livingstone and Russo 2018, licensed under CC BY-NC-SA 4.0.

Experimental stimuli were from the Ryerson Audio-Visual Database of Emotional Speech and Song (Livingstone and Russo, 2018). The stimulus set comprised recordings from 20 actors (10 male, 10 female) producing the utterance “Kids are talking by the door” across multiple emotional conditions (Figure 1; Chege et al., 2026). Each actor expressed six basic emotions (anger, disgust, fear, happiness, sadness, surprise) at both high and low intensity levels, plus one neutral condition, using coordinated facial expressions and vocal prosody. Prior validation by 247 raters using 5-point Likert scales (ranging from ‘very weak’ to ‘very strong’) confirmed that high-intensity conditions achieved “strong” ratings while low-intensity conditions received “weak” ratings (Livingstone and Russo, 2018).

### 2.3 Procedure

Testing occurred in a controlled environment consisting of a sound-attenuated, dimly lit room. Initial eye-tracker calibration employed the TET Calibration package within E-Prime using a nine-point validation protocol. The trial structure began with a blank screen lasting a randomized interval of 600–2,600 ms, followed by a central fixation cross serving as gaze-contingent trial initiation. The video was presented after participants maintained fixation within a 400 × 300 pixel area of interest (AOI) for 400 ms centered on the fixation cross. Following the 3.1-s stimulus presentation, participants completed a two-stage response protocol: first selecting the perceived emotion from a categorical menu via keypress, then rating emotional intensity using a 9-point scale. Response phases were self-paced with no temporal constraints, and participants could initiate rest periods by directing gaze away from the monitor between trials. The experimental stimuli consisted of 260 videos, including 13 conditions (1 neutral + 6 emotions × 2 intensity levels) and 20 trials for each condition. Session duration averaged approximately 45 min.

### 2.4 Questionnaires

To assess individual differences in Autistic traits, four validated self-report measures were administered. The Autism-spectrum Quotient (AQ; Baron-Cohen et al., 2001) is a 50-item inventory covering five domains linked to autism: social skills, attention to detail, attention switching, communication, and imagination. In line with prior evidence suggesting improved reliability in neurotypical populations, the AQ was scored using a four-point Likert scale rather than the original binary system (Stevenson and Hart, 2017). The Broad Autism Phenotype Questionnaire (BAPQ; Hurley et al., 2007) consists of 36 items spanning three subscales: aloof personality, rigid personality, and pragmatic language. Autistic characteristics related to restricted and repetitive behaviors were captured using the Repetitive Behaviors Questionnaire-2A (RBQ-2A; Barrett et al., 2015), a 20-item instrument with four subdomains: repetitive motor movements, rigidity/adherence to routine, preoccupations, and unusual sensory interests. Finally, the Social Responsiveness Scale-2 (SRS-2; Constantino and Gruber, 2012) was used to measure Autism-related patterns in social awareness, cognition, communication, motivation, and restricted interests/repetitive behaviors.

For each participant, raw scores from the AQ, BAPQ, RBQ-2A, and SRS-2 were normalized relative to the maximum possible score for each questionnaire. Descriptive statistics from each measure and the normalized trait composite score are reported in Supplementary Table S1. These standardized scores were then averaged to yield a composite index scaled from 0 to 1 of levels of Autistic traits reflecting Autism traits broadly, calculated as:


$$\text{Trait Composite Score}=(\begin{array}{l} \frac{\text{AQscore}}{\text{AQmax}}+\frac{\text{BAPQscore}}{\text{BAPQmax}}\\ +\frac{\text{RBQscore}}{\text{RBQmax}}+\frac{\text{SRSscore}}{\text{SRSmax}} \end{array})/4$$ (1)

### 2.5 Participant exclusion criteria

Nineteen participants were excluded prior to analysis due to emotion recognition accuracy scores exceeding 2.5 median absolute deviations from the group median (Leys et al., 2013). This criterion identified participants with potentially disengaged performance patterns inconsistent with task demands.

### 2.6 Area of interest definition

**Figure 2:**
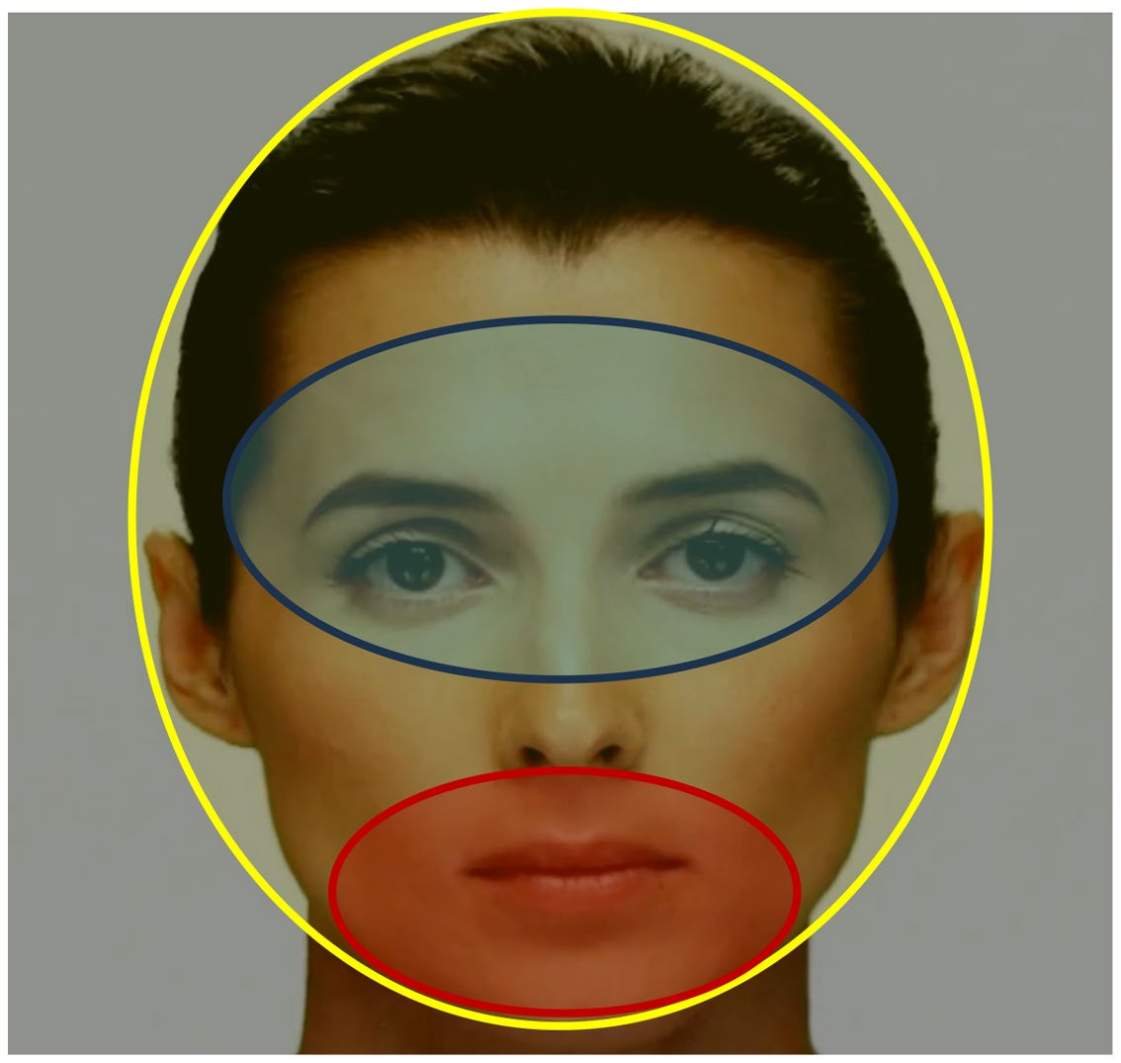
Areas of interest (AOIs) used for gaze analysis. Example stimulus showing the three oval AOIs superimposed on an actor: face (yellow), eye region (blue), and mouth region (red). Stimulus image reproduced from Livingstone and Russo 2018, licensed under CC BY-NC-SA 4.0.

Three oval AOIs were digitally superimposed onto each actor across all trials (Figure 2): eye, mouth, and face regions. The eye AOI was defined with co-vertices positioned at the mid-forehead above the glabella and the nasal bridge midpoint, with vertices extending to the anterior temporal regions lateral to the eye corners. This configuration encompassed the eyes, eyebrows, and lower eyelids. The mouth AOI captured the lips, oral commissures, and mentolabial sulcus, with its minor axis extending from the philtrum midpoint to the chin base and major axis terminating at the bilateral buccal regions. Even though a small AOI offers better specificity, we defined large eye AOIs and mouth AOIs because slight head movement in our video stimuli can render a small AOI less robust. The large AOIs trade specificity, resulting in a small effect, for a reliable and more robust effect. The face AOI encompassed the entire facial region, with vertices positioned at the forehead apex and chin base, and co-vertices at the ear tips. All AOIs were dynamically adjusted to track actor movement throughout each trial, ensuring consistent coverage of the defined facial regions.

### 2.7 Eye-tracking data processing

The Tobii Pro X3-120 Hz system recorded binocular gaze coordinates at 10 Hz intervals on a 1920 × 1080-pixel grid corresponding to monitor resolution. Each trial generated a coordinate sequence mapping participants’ gaze trajectories during video presentation. Missing data points were linearly interpolated for trials with less than 20% data loss; trials exceeding this threshold were excluded from analysis. Fixation durations within AOIs were calculated for each trial, then aggregated by emotional condition. Mean fixation times for each AOI (eye, mouth, face, off-face, off-screen) were computed per emotion and normalized by dividing by the total valid trial duration for each participant. All preprocessing was completed using MATLAB (Mathworks, 2024).

### 2.8 Statistical analysis

The mediation model in this study centers on three key variables: emotion recognition accuracy, levels of Autistic traits, and gaze pattern. Emotion recognition accuracy was calculated as the proportion of correctly identified emotion trials out of all attempted trials. Levels of Autistic traits are measured with the Trait Composite Score (Equation 1). For gaze pattern, two specific metrics were utilized: (A) E-Ratio, defined as the percentage of visual attention directed to the eye area of interest (AOI) out of the total attention to the actor’s face per trial, (B) M-Ratio, reflecting the proportion of attention allocated to the mouth AOI during each trial. All correlation analyses were run using either Spearman’s rho (*ρ*) or Pearson’s r. If any variable in a correlation analysis has skewness greater than 1 or less than −1 (boundary included), Spearman’s rho was used. Otherwise, we used Pearson’s r for analyses (see Supplementary Table S2 for the skewness of all variables). All statistical analyses were conducted in JASP (Team, 2025). All exploratory analyses will be corrected for multiple comparisons using Benjamini-Hochberg false discovery rate (*α* = 0.05, *Q* = 0.15).

### 2.9 Preliminary validation and data preparation

Prior to conducting the primary analyses, we performed validation checks to confirm stimulus effectiveness. First, we examined whether the intensity salience manipulation significantly affected participants’ gaze patterns. E-Ratio and M-Ratio were separated for high and low intensity trials, and a paired *t*-test was performed. If no significant difference emerged between intensity conditions, data would be collapsed across salience levels for subsequent analyses.

Next, behavioral responses were examined to verify that participants accurately identified the intended emotional categories. Mean emotion classification accuracy was computed for each participant and experimental condition. This validation procedure confirmed that emotional content was reliably recognizable.

### 2.10 Primary analysis strategy

Following these preliminary steps, we adopted a hierarchical analytical approach to examine relationships between gaze fixation patterns, emotion recognition accuracy, and levels of Autistic traits. This strategy proceeded systematically from global to specific levels of analysis.

We first examined correlations using aggregated scores across all emotions to identify overarching patterns. Three sets of correlations were conducted: (A) gaze fixation M-Ratio/E-Ratio and levels of Autistic traits composite scores, (B) M-Ratio/E-Ratio and emotion recognition accuracy, and (C) Autistic traits and emotion recognition accuracy. Most correlations performed are Pearson correlation; however, if the skewness is greater than one, then Spearman correlation is used instead. This global approach established whether meaningful relationships existed at the general level before proceeding to more specific analyses.

Following the identification of significant correlations in the global analysis, we conducted repeated-measures ANOVA to examine within-group differences in gaze patterns across the six basic emotions and the neutral condition. This analysis established whether gaze allocation patterns vary significantly across different emotional expressions, thereby supporting subsequent emotion-specific investigations.

Based on significant findings from the global analysis and confirmation of emotion-specific gaze pattern differences, we proceeded to examine correlations for individual emotions. This targeted approach focused on the specific emotions and measures that showed significant associations in the global analysis, comprising separate correlations for each of the six basic emotions and the neutral condition. For emotion-specific analyses (from section 3.3.2 to 3.3.4), we adopted the Benjamini-Hochberg false discovery rate (FDR) approach (Q = 0.15) to combat the inflation of type 1 error.

The primary objective of this analytical framework was to confirm that levels of Autistic traits correlate with recognition accuracy and to identify associations between Autistic traits, emotion recognition performance, and gaze allocation to specific facial regions. Analyses focused particularly on eye and mouth AOI fixation durations and the eye-to-mouth fixation ratio.

## 3 Results

### 3.1 Preliminary validation and data preparation

#### 3.1.1 Salience manipulation analysis

**Figure 3:**
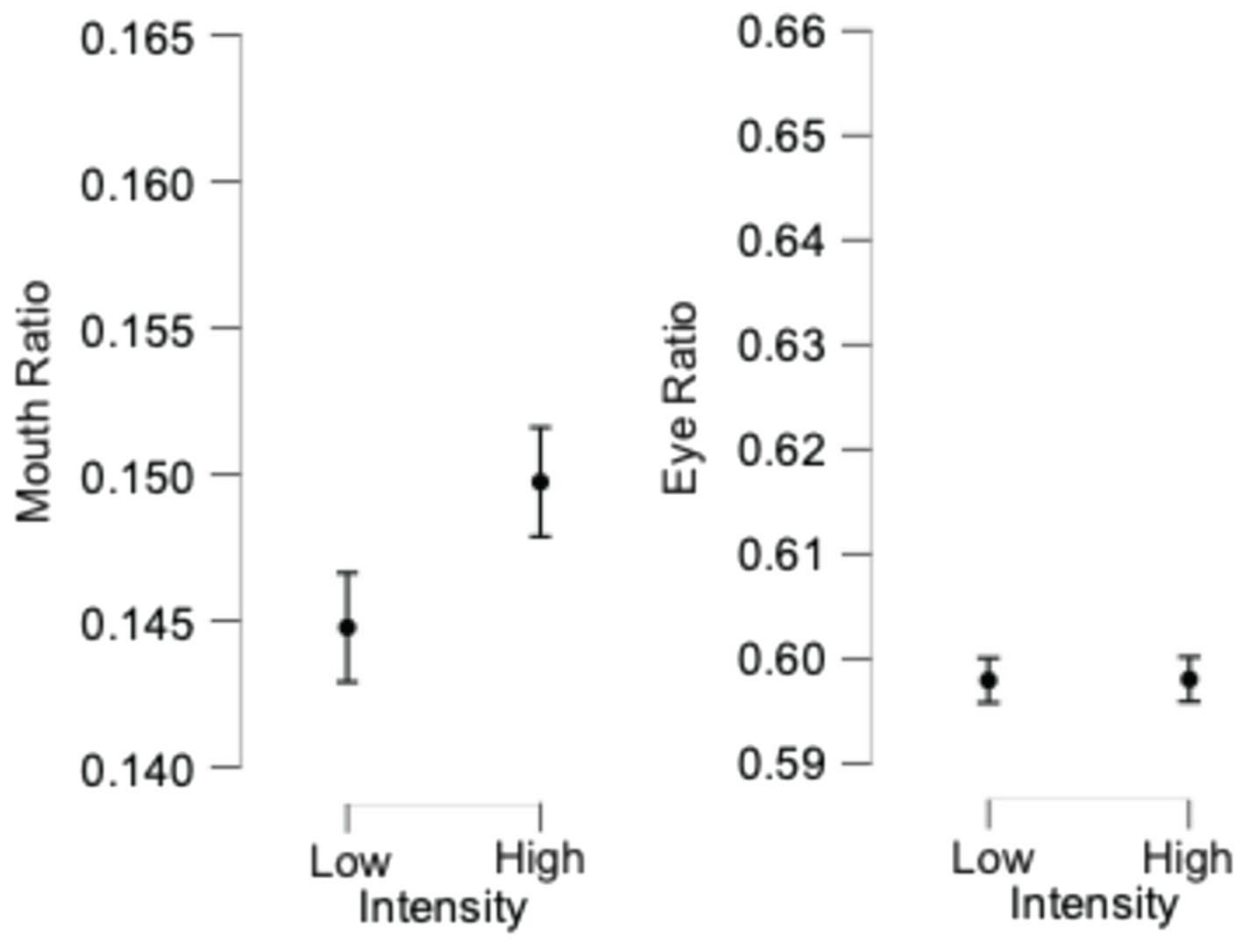
Compare low vs. high intensity conditions for mouth ratio and eye ratio. Left: *T*-test between low and high intensity mouth AOI fixation time. Right: *T*-test between low and high intensity eye AOI fixation time. Neither reached statistical significance. Error bars represent standard error.

Initial analyses examined the impact of intensity salience manipulation on participants’ gaze patterns across the M-Ratio and E-Ratio metrics. Paired *t*-tests revealed no significant differences between high and low intensity conditions for either gaze metric [*t*_mouth_(80) = −1.88, *p* = 0.06; *t*_eyes_(80) = 1.26, *p* = 0.21], as shown in Figure 3. Data were therefore collapsed across salience levels for subsequent analyses.

#### 3.1.2 Emotion recognition validation

**Figure 4:**
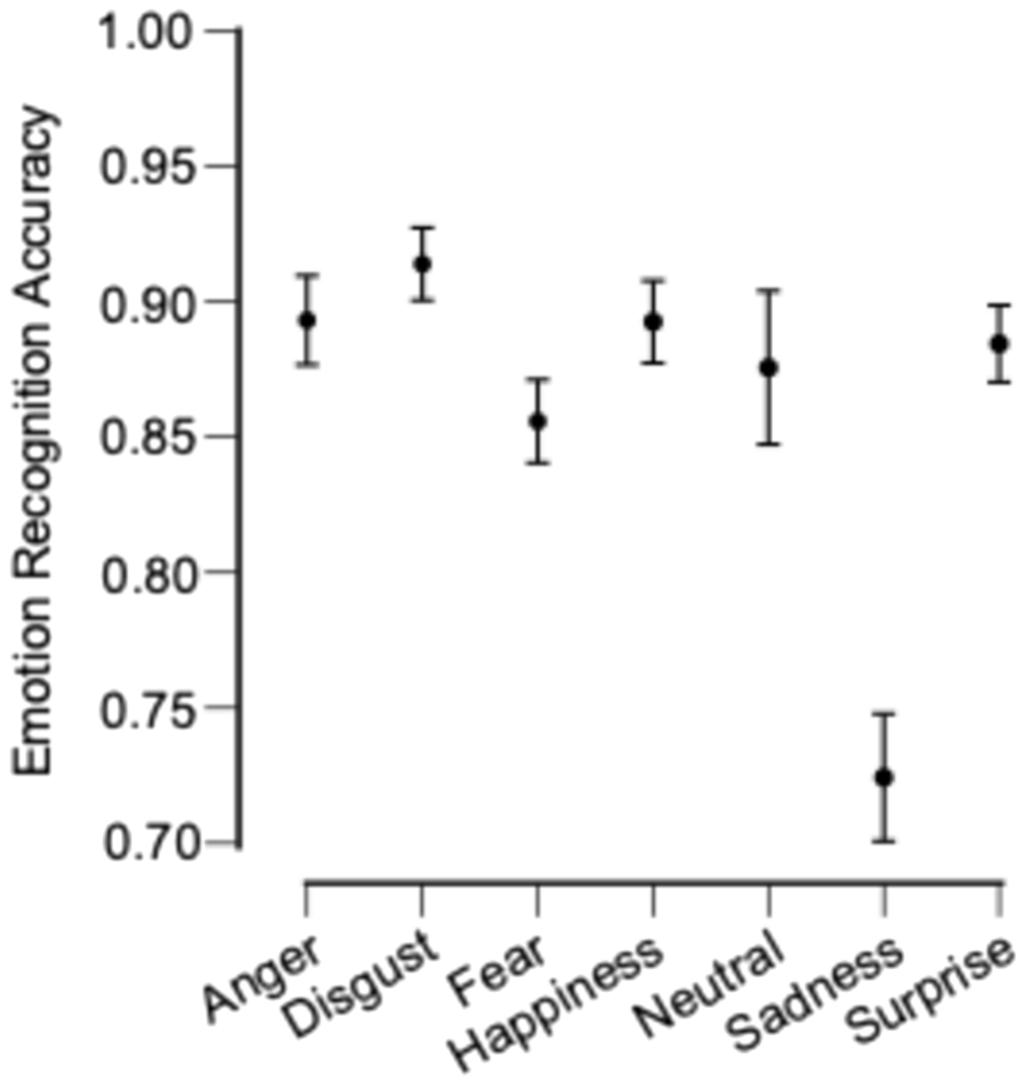
Emotion recognition accuracy between different emotions. Participants were able to recognize emotions presented in the stimuli, although they particularly struggled with recognizing sadness. Error bars represent standard error.

Initial validation analyses confirmed that the emotional stimuli were effectively recognizable by participants. However, sadness was recognized substantially less consistently compared to all other emotions and was therefore excluded from subsequent analyses (Figure 4).

### 3.2 Global cross-emotion analysis

#### 3.2.1 Levels of Autistic traits and emotion recognition accuracy

**Figure 5:**
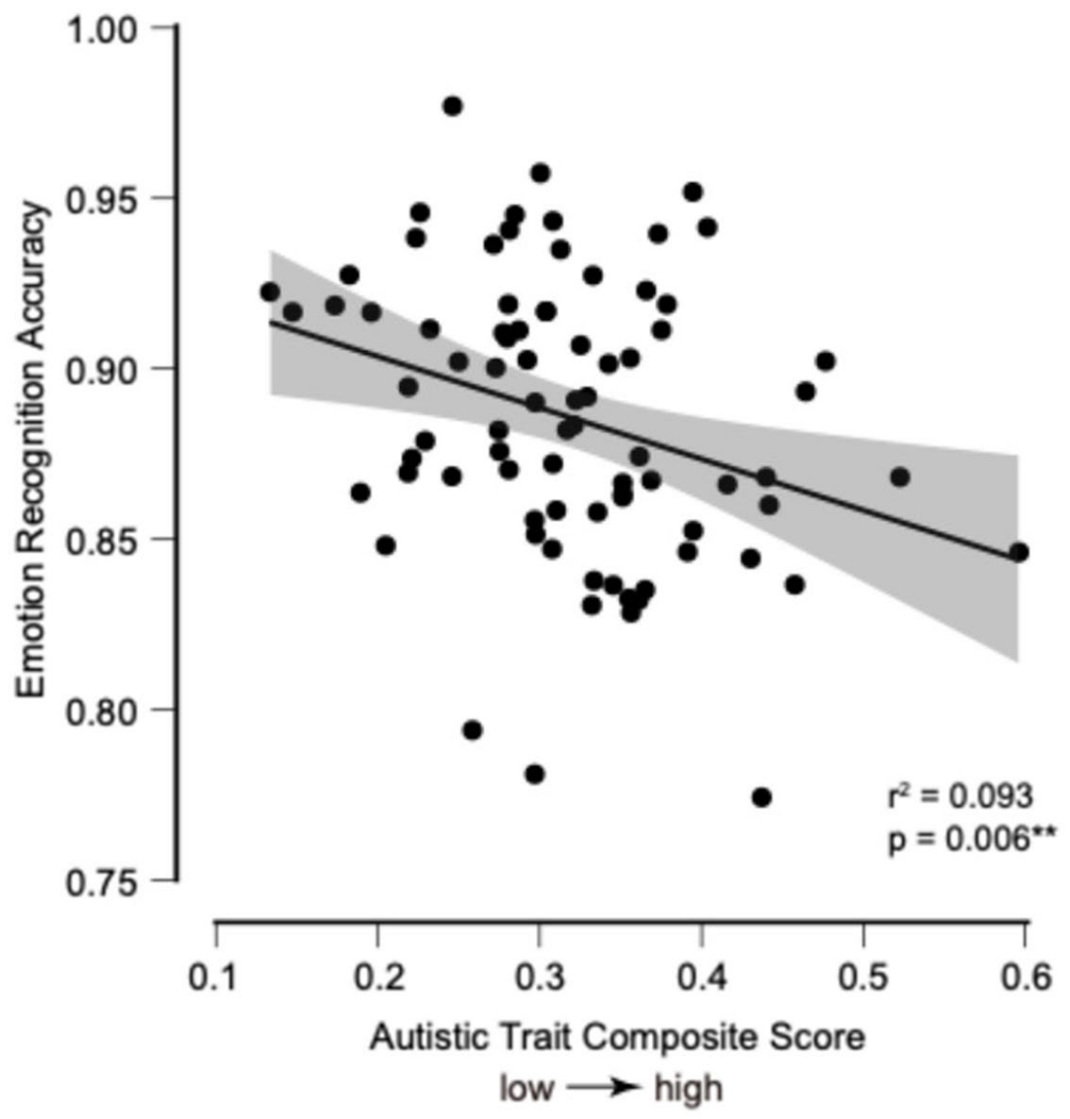
Higher levels of Autistic traits composite scores associated with lower emotion recognition accuracy. Greater autistic trait composite scores are associated with greater emotion recognition difficulties.

We first assessed the association between levels of Autistic traits and emotion recognition accuracy collapsed across all emotions. Pearson correlation analysis revealed a significant negative correlation between Autistic Trait Composite Scores and overall emotion recognition accuracy [*r*(79) = −0.30, *p* = 0.006, Figure 5]. Higher levels of Autistic traits were associated with reduced emotion recognition performance.

#### 3.2.2 Gaze pattern and emotional accuracy

**Figure 6:**
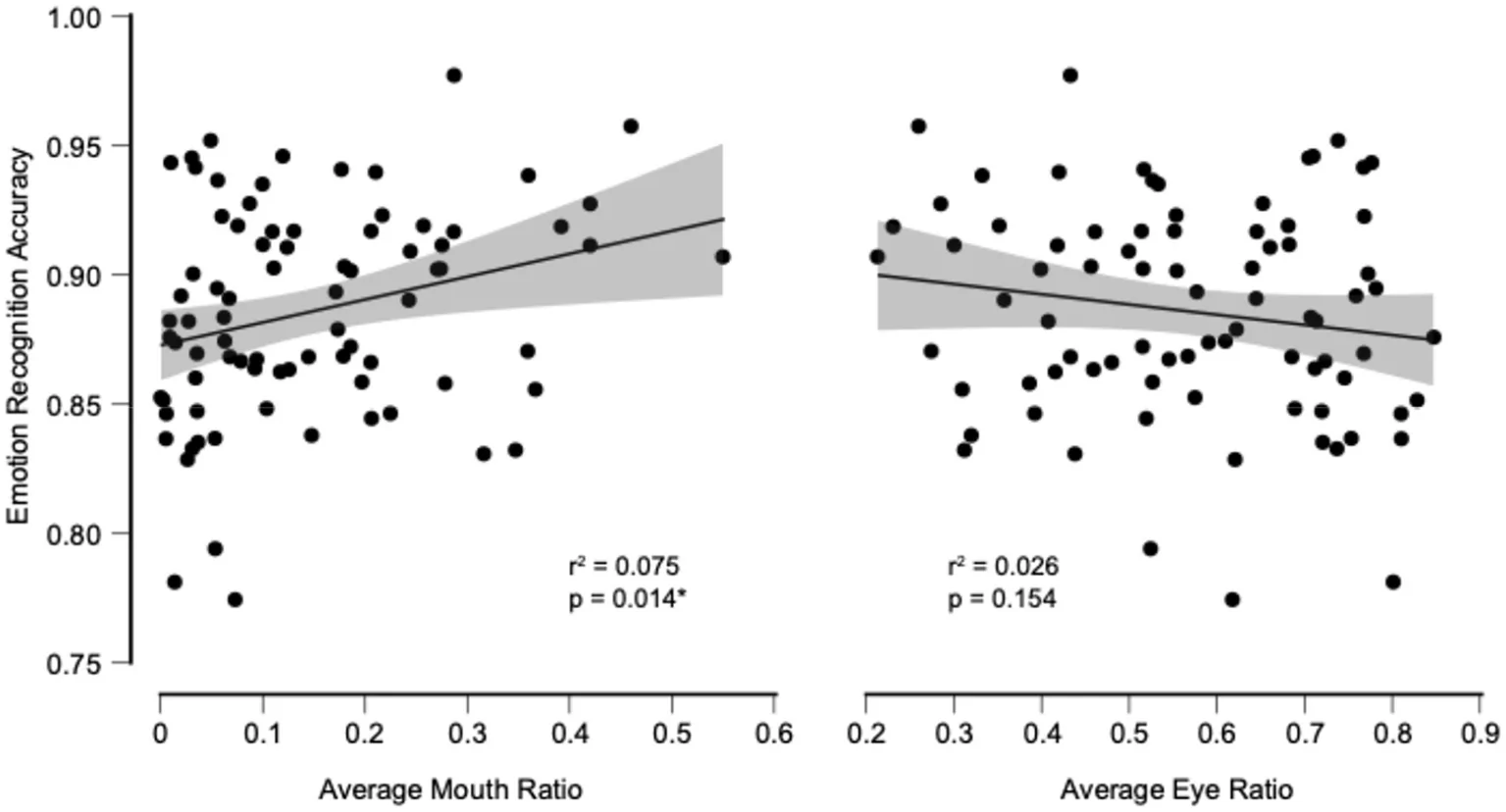
Correlations between overall emotion recognition accuracy and average eye ratio and mouth ratio. Average mouth ratio positively correlated with overall emotion recognition accuracy, whereas average eye ratio did not.

To establish our proposed mediation model, we next investigated the relationships between gaze patterns and overall emotion recognition accuracy. The analysis revealed that average M-Ratio positively correlated with overall recognition accuracy [*r*(79) = 0.27, *p* = 0.014], whereas average E-Ratio was negatively associated with overall accuracy, but this association failed to reach statistical significance [*r*(79) = −0.16, *p* = 0.154], as shown in Figure 6.

#### 3.2.3 Levels of Autistic traits and gaze patterns

**Figure 7:**
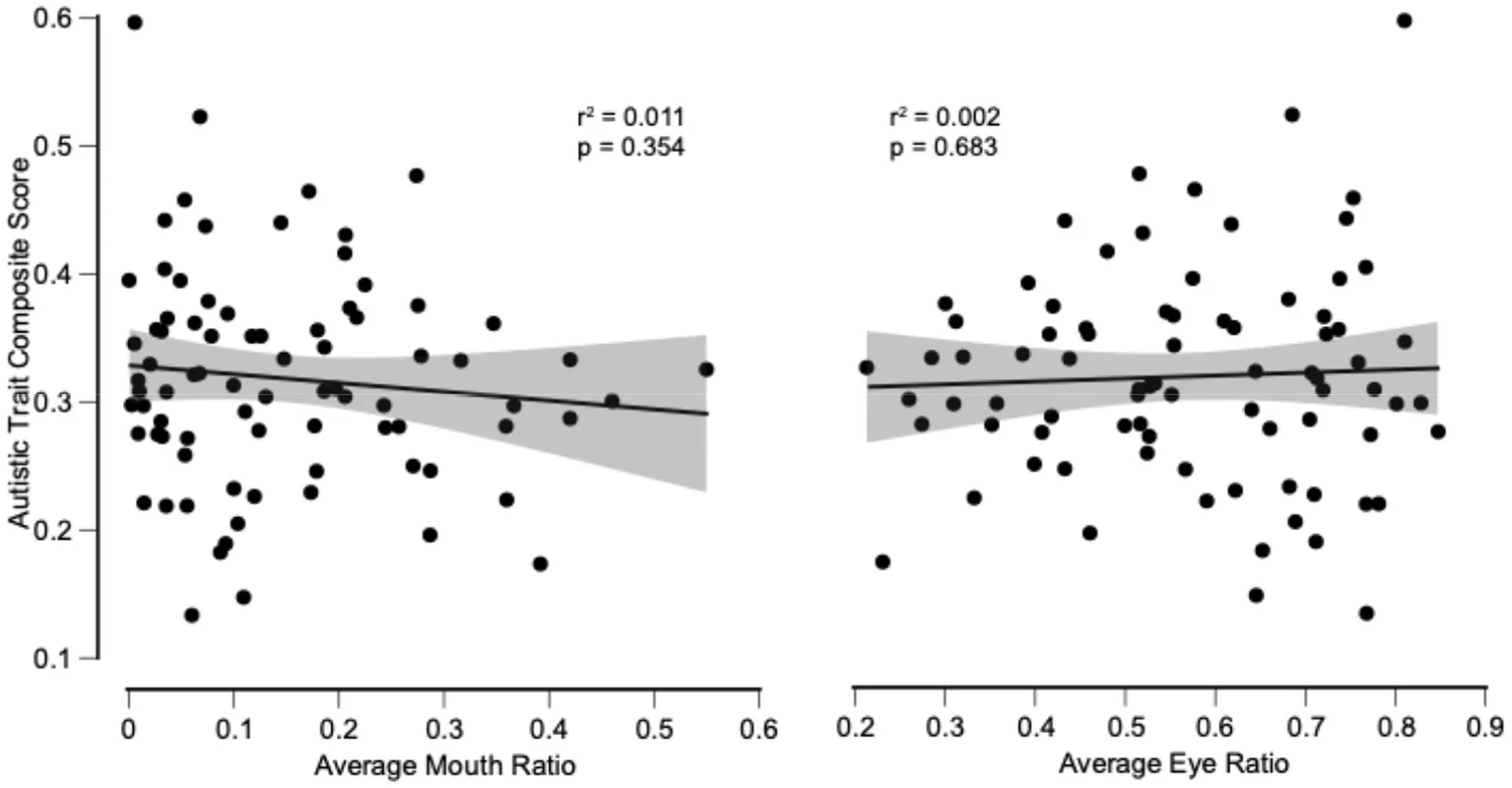
Correlations between average gaze patterns and Autistic Trait Composite Scores. We found no associations between average gaze patterns and autistic trait composite scores.

We then examined correlations between levels of Autistic traits and gaze patterns. No significant relationship was observed between levels of Autistic traits and average M-Ratio [*r*(79) = −0.10, *p* = 0.35] or E-Ratio [*r*(79) = −0.05, *p* = 0.68], shown in Figure 7.

Given the lack of association between levels of Autistic traits and eye gaze patterns, we were unable to conduct the planned mediation analysis between Autistic traits, eye gaze patterns, and emotion recognition accuracy. However, Autistic traits can be parsed into two domains, social difficulties and repetitive behaviors and interests. As exploratory analyses, we ran correlations with SRS and RBQ, which capture the two domains, respectively. RBQ scores correlated with gaze fixation pattern to mouth region [*ρ*(79) = −0.307, *p* = 0.005] but not the eye region [*ρ*(79) = −0.097, *p* = 0.390], and it correlated with overall recognition accuracy [*ρ*(79) = −0.350, *p* = 0.001], whereas SRS score did not correlate with either gaze patterns [eye region: *r*(79) = −0.060, *p* = 0.596; mouth region: *r*(79) = 0.002, *p* = 0.988] or overall accuracy [*r*(79) = −0.197, *p* = 0.077]. We then proceeded with the only viable mediation analysis, investigating if the correlation between RBQ and recognition accuracy is mediated by fixation to the mouth. With a 500-bootstrap resampling, we failed to detect a mediation effect with mouth fixation (Supplementary Table S4; indirect effect: *Z* = −1.55, *p* = 0.121).

### 3.3 Emotion-specific analyses

#### 3.3.1 Emotion-specific gaze patterns

**Figure 8:**
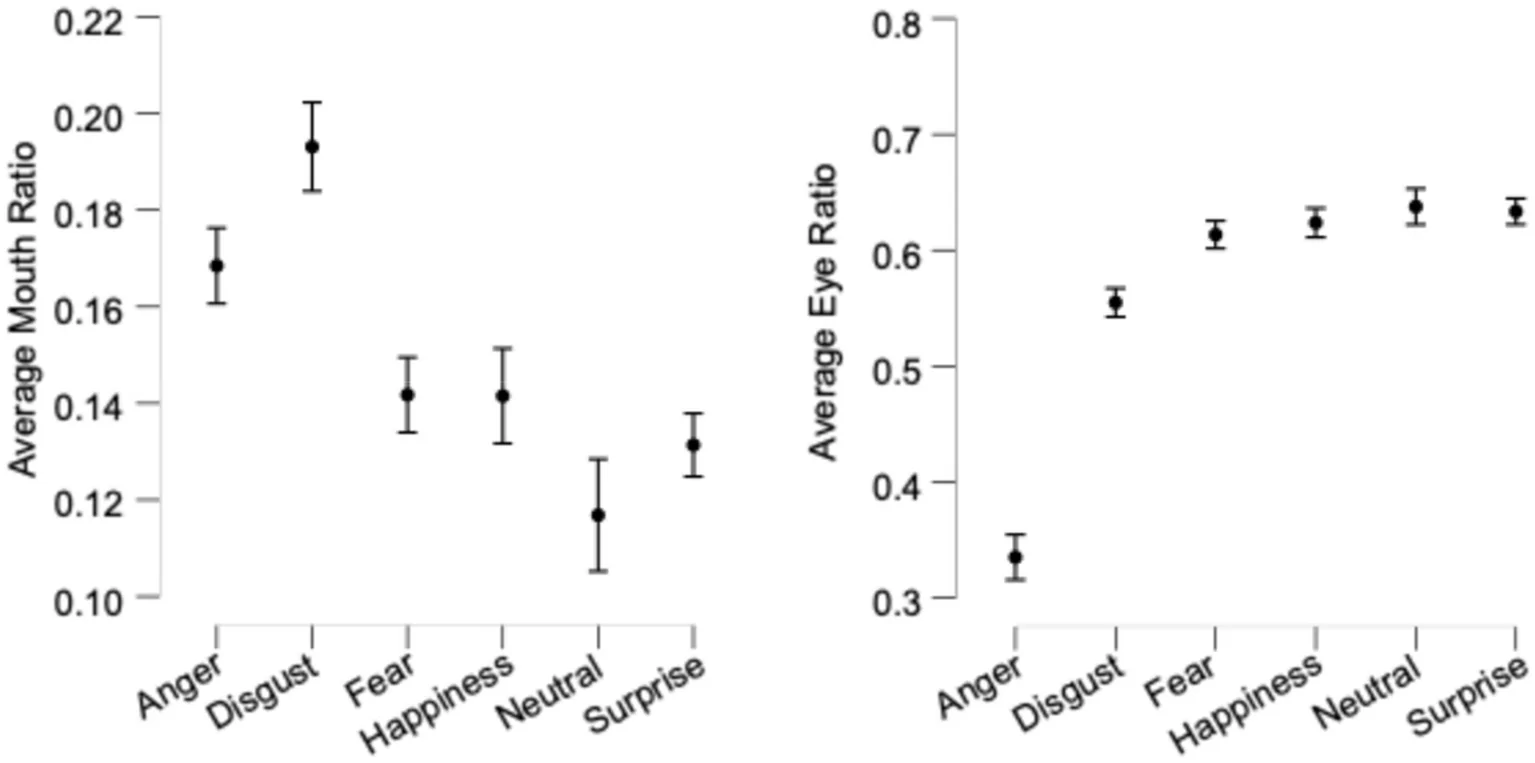
Proportion of time spent on the mouth (left) and eyes (right) relative to face. Black dots indicate the mean values. Error bars represent standard error.

Before proceeding to exploratory emotion-specific correlational analyses, we examined whether participants’ gaze patterns varied significantly across different emotions. This analysis established if emotion-specific investigations were warranted based on the differential gaze allocation patterns across emotional expressions (Figure 8).

We conducted a repeated-measures ANOVA to examine participants’ gaze fixation patterns across the six emotions, using the proportion of time spent fixating on the mouth and eye regions as indices of gaze behavior. The analysis revealed significant differences in gaze patterns across emotions for both M-Ratio [*F*(5,400) = 37.33, *p* < 0.001, *η*^2^ = 0.04] and E-Ratio [*F*(5,400) = 272.28, *p* < 0.001, *η*^2^ = 0.27]. These findings confirmed that participants differentially allocated their visual attention when viewing distinct emotional expressions, thereby supporting the rationale for conducting subsequent emotion-specific correlational analyses.

#### 3.3.2 Levels of Autistic traits and individual emotion recognition

In our exploratory analyses, we examined correlations between levels of Autistic traits and recognition accuracy for each individual emotion, matching the global analysis that revealed a significant negative correlation between Autistic traits and overall emotion recognition accuracy.

**Figure 9:**
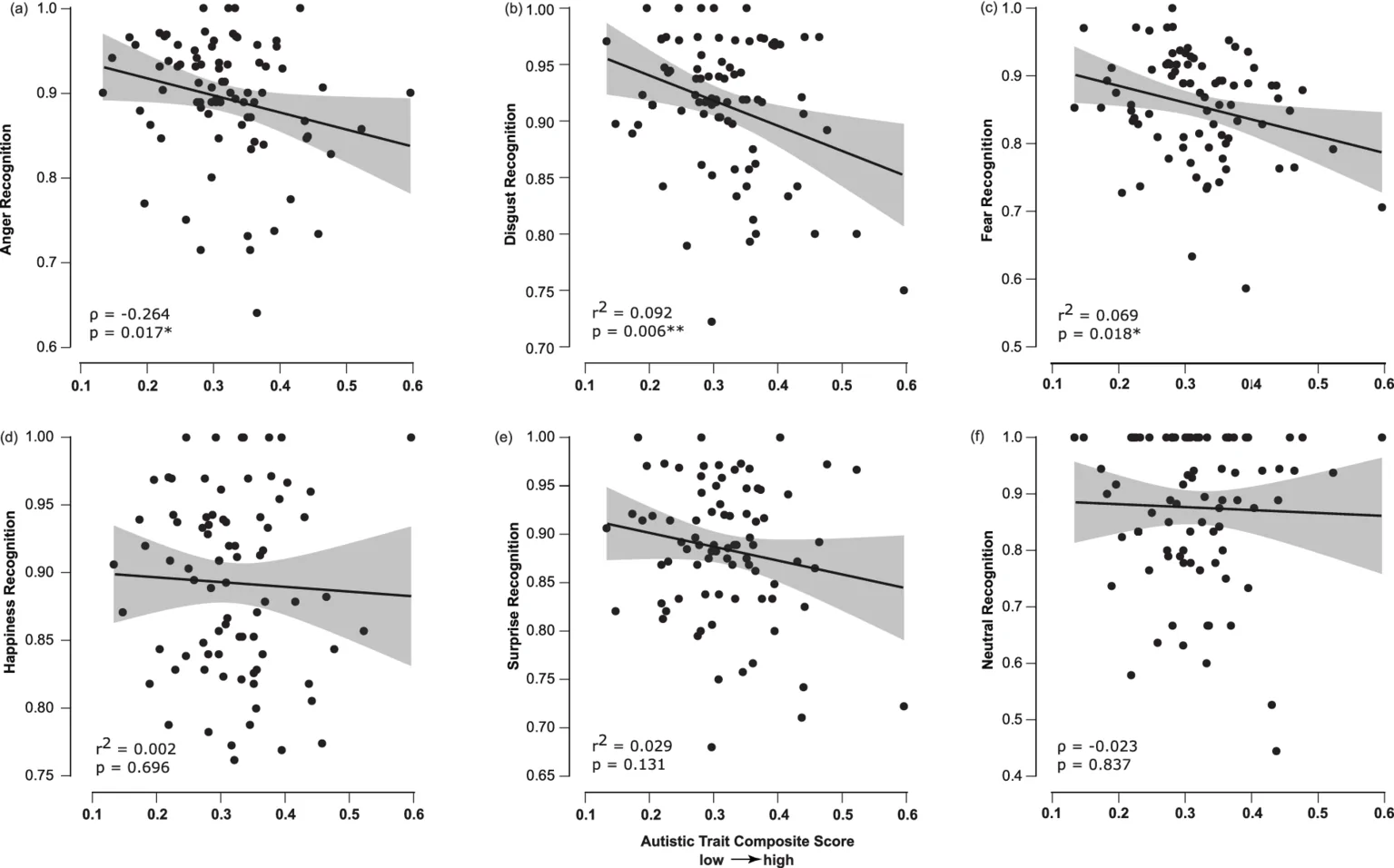
Correlation between each emotion’s recognition accuracy and levels of autistic traits. Autistic Trait Composite Score is negatively correlated with the recognition of anger, disgust, and fear. It is not correlated with the recognition of happiness, surprise, and neutral emotion.

Level of Autistic traits showed significant negative correlations with recognition accuracy for the negative emotions: anger [*ρ*(79) = −0.264, *p* = 0.017], disgust [*r*(79) = −0.304, *p* = 0.006], and fear [*r*(79) = −0.262, *p* = 0.018]. However, no significant correlations emerged between level of Autistic traits and recognition accuracy for happiness [*r*(79) = −0.044, *p* = 0.696], surprise [*r*(79) = −0.169, *p* = 0.131], or neutral [*ρ*(79) = −0.023, *p* = 0.837]. These findings indicate that higher levels of Autistic traits composite score are associated with specific difficulties in recognizing negative emotions (Figure 9).

#### 3.3.3 Gaze patterns and individual emotion recognition

Following the global analysis that revealed M-Ratio to be positively correlated with overall emotion recognition, we examined correlations between emotion-specific gaze patterns and recognition accuracy for each corresponding emotion.

**Figure 10:**
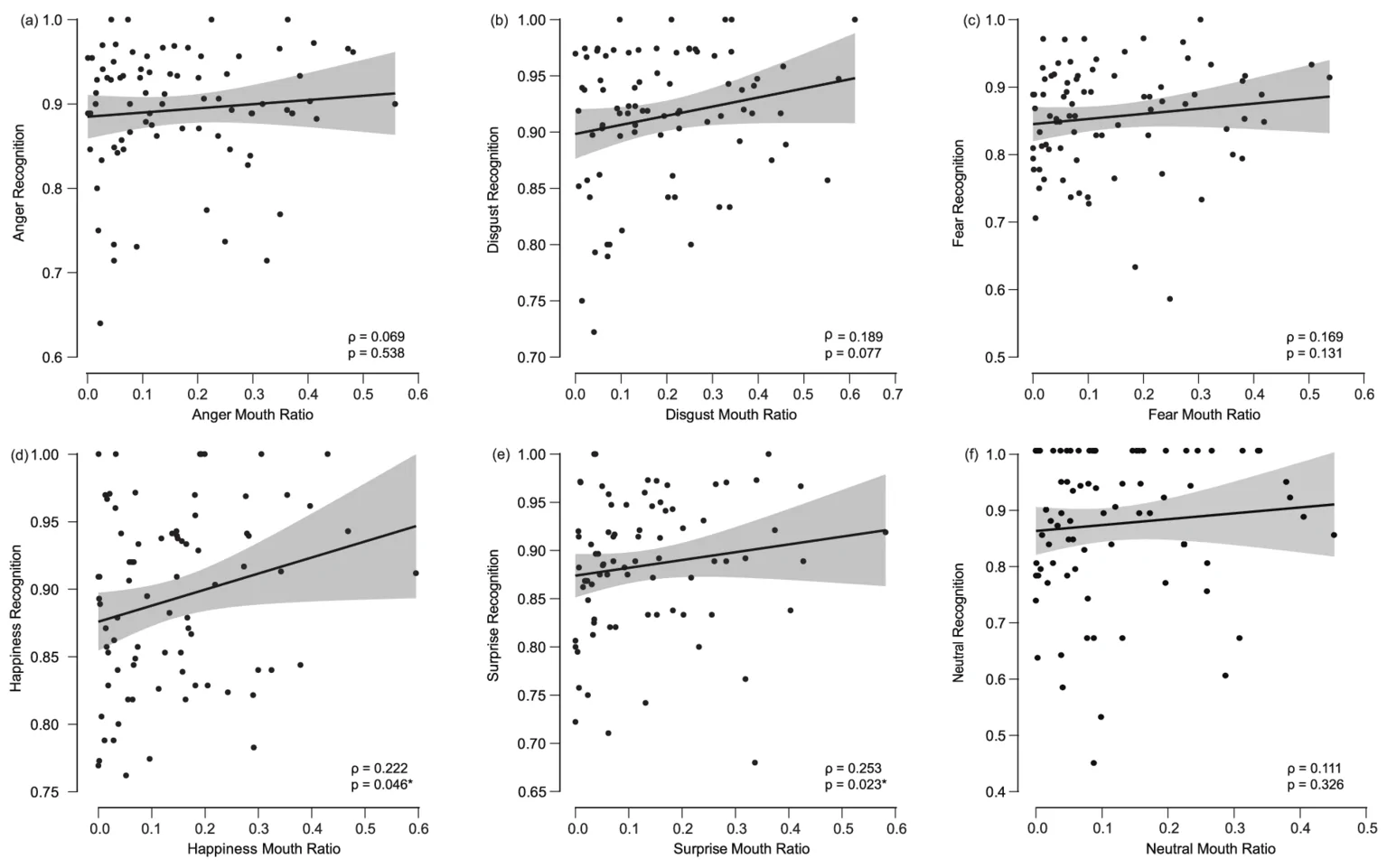
Recognition accuracy of each emotion and mouth ratio. Only the happiness and surprise accuracy are positively correlated with their own mouth ratio. Other accuracy and mouth ratio pairs did not reach significance.

Emotion-specific M-Ratio showed significant positive correlations with recognition accuracy for happiness [*ρ*(79) = 0.222, *p* = 0.046] and surprise [*ρ*(79) = 0.253, *p* = 0.023]. No significant correlations were found between M-Ratio and recognition accuracy for anger [*ρ* (79) = 0.069, *p* = 0.538], disgust [*ρ*(79) = 0.198, *p* = 0.077], fear [*ρ*(79) = 0.169, *p* = 0.131], or neutral [*ρ*(79) = 0.111, *p* = 0.326], shown in Figure 10. These results suggest that greater attention to the mouth region is beneficial for the recognition of happiness and surprise. Consistent with the global analysis showing no significant correlation between E-Ratio and overall recognition accuracy, emotion-specific E-Ratio did not significantly correlate with recognition accuracy for any individual emotion.

#### 3.3.4 Levels of Autistic traits and individual emotion gaze patterns

We examined correlations between levels of Autistic traits and emotion-specific gaze patterns for each emotion, following the global analysis that showed no significant correlations between Autistic traits and overall gaze patterns. No significant correlations emerged between levels of Autistic traits and M-Ratio or E-Ratio for any individual emotion (Supplementary Table S3).

## 4 Discussion

Difficulties in emotion recognition that are well-documented in clinically diagnosed Autistic individuals have also been observed in the broader population in relation to levels of Autistic traits (West et al., 2020, Standiford and Hsu, 2026, Reed et al., 2021). The mechanisms underlying this association in the broader population are poorly understood. This study examined one possible mechanism by investigating whether gaze patterns to specific facial regions mediate the relationship between Autistic traits and emotion recognition. Consistent with previous research, our data demonstrate that higher levels of Autistic traits were associated with lower overall recognition accuracy, and greater mouth attention was associated with higher recognition accuracy. However, gaze patterns were not correlated with levels of Autistic traits (Figure 7), and thus our proposed mediation models were not supported. Emotion-specific effects were observed that provide a potential explanation for these findings, where levels of Autistic traits related only to negative emotions (fear, anger, disgust), whereas time spent attending to the mouth was associated with higher recognition of happiness and surprise (Figure 10).

In evaluating the direct association central to our proposed mediation model, our findings confirmed the established relationship between levels of Autistic traits and emotion recognition accuracy; individuals with higher levels of Autistic traits have lower recognition accuracy for negative emotions but not for positive emotions (Ji et al., 2025; Schnitzler et al., 2024; Dollion et al., 2022; Masoomi et al., 2025; Yeung, 2022; Tamas et al., 2024).

We hypothesized that this effect would be driven by changes in patterns of gaze to the eyes and mouth associated with higher levels of Autistic traits. This hypothesis was based on previous studies in Autism that reported reduced time spent looking at the eyes, coupled with research suggesting negative emotions in particular are more effectively identified by information from the eyes (Kim et al., 2022; Šķilters et al., 2024; Chen et al., 2015; Calvo and Nummenmaa, 2016). Thus, reduced gaze to the eyes would result in missing cues that are critical for decoding negative emotions (Stuart et al., 2023; Trevisan et al., 2017; Hadjikhani et al., 2017). Contrary to expectations, our data revealed no correlation between levels of Autistic traits and eye gaze patterns, suggesting that visual attention to different facial regions does not mediate the relationship between emotion recognition difficulties and levels of Autistic traits. This outcome does align with recent reviews noting that studies of Autistic traits in the broader population often observe weaker or more variable associations between Autistic traits and gaze behavior than observed in diagnosed Autism (Stuart et al., 2023; Nummenmaa et al., 2012; Bagherzadeh-Azbari et al., 2022). The reduction in time spent looking at the eyes in Autism has been attributed to a hyperarousal response during eye contact (Trevisan et al., 2017), but it is uncertain whether this response extends beyond diagnosed Autism into individuals in the broader population who exhibit high levels of Autistic traits. Our data shed light on a more complex system showing rigidity and repetitive behaviors, one of the two major domains of Autistic traits, reliably predict reduced fixation toward mouth and prolonged fixation toward eyes whereas the social aspect of Autistic traits correlated with neither. Coupled with our findings that the pattern of emotion recognition difficulties observed in Autism does extend to individuals with high levels of Autistic traits in the broader population, this provides evidence that gaze patterns do not appear to play a mechanistic role in the relationship between levels of Autistic traits and emotion recognition ability in the non-Autistic population, despite the association with rigidity and repetitive behavior.

A different pattern of associations was seen with levels of Autistic traits, patterns of eye gaze, and emotions of happiness and surprise. Our results demonstrate a positive association between greater fixation on the mouth region and recognition of happiness and surprise, which is consistent with other work on dynamic facial expressions (Calder et al., 2000; Beaudry et al., 2014). Positive emotions like happiness are more closely associated with mouth cues than eye cues (Kim et al., 2022; Šķilters et al., 2024; Chen et al., 2015; Calvo and Nummenmaa, 2016), though findings are more nuanced for surprise, which is more balanced between the mouth and eyes in terms of emotional information (Calvo and Nummenmaa, 2016; Kim et al., 2022; Eisenbarth and Alpers, 2011), though previous findings report that lip movements are especially diagnostic for these expressions (Schurgin et al., 2014; Wegrzyn et al., 2017). The increase in time looking at the mouth relative to the eyes in Autism may then explain the lack of difficulties in recognizing positive emotions relative to negative emotions. However, in our data, the level of Autistic traits was not associated with time spent looking toward the mouth, yet the specific association between the level of Autistic traits and difficulty recognizing negative emotions remains. In addition, contrary to previous evidence, we found that fixation to the eyes does not correlate with recognition of negative emotions. This can also be attributed to the lack of clinical samples due to the potential moderating role of ASD differentiating the relationship between emotion recognition and gaze patterns (e.g., Wieckowski and White, 2017) as gaze pattern of Autistic individuals might be qualitatively different than that of non-Autistic people. Taken together, our data provide further evidence that patterns of eye gaze in Autism may not extend to individuals with high levels of Autistic traits in the broader population, and that eye gaze patterns may not underlie the emotion-recognition difficulties associated with high levels of Autistic traits observed here and in previous studies. Other lines of research have suggested alternative explanations regarding the relationship between Autism and emotion recognition that might explain this relationship at the trait level, such as the alexithymia theory. Alexithymia is a trait characterizing people’s impaired ability to describe their own emotions. The alexithymia theory is built on the premises that (1) people with high levels of alexithymia often have trouble recognizing facial emotions of others in addition to their own emotions and (2) there is an extensive overlap between Autistic people and high-level of alexithymia. It argues that the observation that Autistic people’s difficulties recognizing emotions are not due to Autism itself but rather the high levels of alexithymia (Cook et al., 2013; Heaton et al., 2012). Other hypotheses have attempted to mechanistically explain emotion recognition difficulties among Autistic individuals, such as the double empathy problem (Cheang et al., 2025; Milton et al., 2023), and previous work has suggested that emotion recognition difficulties vary based on numerous task parameters, such as the presence of contextual cues (Metcalfe et al., 2019).

Emotional cues are offered through both facial expression and auditory prosody (such as pitch and volume). Although we implemented dynamic facial stimuli, the laboratory setting lacks the ecological validity and complexity of real-world social interactions, potentially affecting how naturally gaze behaviors manifest in relation to Autistic traits. This represents a potential inherent disadvantage of using dynamic facial stimuli in controlled laboratory environments. Real-world social interactions may involve multifaceted contextual cues, including body language, environmental factors, social expectations, and the dynamic interplay of conversation, all of which might influence gaze patterns and emotional processing. In contrast, our paradigm presented isolated facial expressions on a computer screen, possibly removing the rich social context that typically accompanies emotion recognition in everyday life. This decontextualized presentation may have reduced the salience of emotional cues that could normally trigger differential gaze behaviors between individuals with varying levels of Autistic traits. It is worth noting that much work in the past used visual-only facial expressions to study gaze patterns to emotional stimuli. Though the addition of auditory prosody creates a more ecologically valid stimulus than uni-sensory stimuli alone, this complicates direct comparisons with uni-sensory findings. Additionally, our sample was over three-quarters women, which matches neither the gender ratio of the general population nor the Autistic population, which should be considered when attempting to generalize these findings to either population.

## 5 Conclusion

In summary, this study reveals that Autistic traits in neurotypical adults are reliably associated with poorer recognition of negative facial emotions, yet gaze patterns toward the eyes or mouth do not explain these deficits. Instead, mouth-centric attention emerged as a stronger predictor of accuracy, particularly for positive emotions. Importantly, the anticipated mediation model between Autistic traits, gaze allocation, and emotion recognition was unsupported, highlighting the need to reconsider established theories and to explore alternative mechanisms, such as feature-based processing and multimodal cues, for emotion recognition difficulties. Greater representation of clinically diagnosed Autistic participants and ecologically valid paradigms in future work will be vital for clarifying these complex relationships.

## Data Availability

The datasets presented in this study can be found in online repositories. The names of the repository/repositories and accession number(s) can be found at: https://osf.io/cqf5s/overview?view_only=1f6c1417da3f496d97d9af98f77bb31e.
