# Supplementary material for: Testing the relationships between facial emotion recognition, levels of Autistic traits, and eye gaze patterns
Source: Front Hum Neurosci. 2026 Aug 24;20:1824976. doi: 10.3389/fnhum.2026.1824976 (PMC13547462; doi:10.3389/fnhum.2026.1824976)
Supplement: Supplementary file 1 [file Supplementary_file_1.docx]

**Supplemental Material**

**Table S1. Descriptive statistics of Autistic trait measures.**

| Measure | Mean(SD) | Range | Skewness(SD) | Kurtosis(SD) |
| --- | --- | --- | --- | --- |
| AQ | 110(12.4) | 80-139 | 0.013(0.27) | -0.038(0.53) |
| BAPQ | 102(20.1) | 45-169 | 0.318(0.27) | 1.86(0.53) |
| RBQ | 31.3(7.4) | 83-167 | 1.370(0.27) | 2.39(0.53) |
| SRS | 117(18) | 22-60 | 0.522(0.27) | 0.191(0.53) |
| Composite | 0.3(0.083) | 0.13-0.60 | 0.459(0.27) | 0.925(0.53) |

**Note*.	AQ – Autism-spectrum Quotient, BAPQ – Broad Autism Phenotype Questionnaire, RBQ – Repetitive Behavior Quotient, SRS – Social Responsiveness Scale, SD – standard deviation. N = 81.

**Table S2. Skewness of Variables**

| Variable | Skewness | SE |
| --- | --- | --- |
| Average Mouth Ratio | 0.9791 | 0.2673 |
| Average Eye Ratio | -0.2758 | 0.2673 |
| Anger Mouth Ratio  Anger Eye Ratio | 0.7433  0.2682 | 0.2673  0.2673 |
| Disgust Mouth Ratio  Disgust Eye Ratio | 0.782  0.0006 | 0.2673  0.2673 |
| Fear Mouth Ratio  Fear Eye Ratio | 1.0435  -0.5041 | 0.2673  0.2673 |
| Happiness Mouth Ratio  Happiness Eye Ratio | 1.1154  -0.2864 | 0.2673  0.2637 |
| Neutral Mouth Ratio  Neutral Eye Ratio | 1.1477  -0.2247 | 0.2673  0.2673 |
| Sadness Mouth Ratio  Sadness Eye Ratio | 0.7944  -0.1959 | 0.2673  0.2673 |
| Surprise Mouth Ratio  Surprise Eye Ratio | 1.2455  -0.3622 | 0.2673  0.2673 |
| Overall Recognition accuracy | -0.3535 | 0.2673 |
| Anger Accuracy | -1.1756 | 0.2673 |
| Disgust Accuracy | -0.9513 | 0.2673 |
| Fear Accuracy | -0.8213 | 0.2673 |
| Happiness Accuracy | -0.1239 | 0.2673 |
| Neutral Accuracy | -1.0815 | 0.2673 |
| Sadness Accuracy | -0.8598 | 0.2673 |
| Surprise Accuracy | -0.6613 | 0.2673 |

*Note.* Any correlational analysis involving at least one variable whose skewness is either above or below 1 will use Spearman’s rho. Others will use Pearson’s r. SE = Standard error of the mean.

**Table S3. Correlations between Autistic Trait Composite Score and Mouth Ratio for Each Emotion**

| Emotion | Correlation | p-value |
| --- | --- | --- |
| Anger Mouth Ratio  Anger Eye Ratio | r = -0.10  r = 0.019 | 0.37138  0.86723 |
| Disgust Mouth Ratio  Disgust Eye Ratio | r = -0.11  r = 0.07 | 0.32826  0.50788 |
| Fear Mouth Ratio  Fear Eye Ratio | ρ = -0.07  r = 0.05 | 0.54665  0.66781 |
| Happiness Mouth Ratio  Happiness Eye Ratio | ρ = -0.10  r = 0.05 | 0.38479  0.65389 |
| Neutral Mouth Ratio  Neutral Eye Ratio | ρ = 0.04  r = 0.03 | 0.71202  0.81526 |
| Surprise Mouth Ratio  Surprise Eye Ratio | ρ = -0.06  r = 0.03 | 0.61656  0.76161 |

*Note*. Autistic Trait Composite Score did not correlate with mouth gaze pattern for any emotions.

**Table S4. *Mediation Analysis with RBQ, Mouth Fixation, and Overall Recognition Accuracy***

| Effect | Z | p |
| --- | --- | --- |
| Indirect | -1.55 | 0.121 |
| Direct | -2.77 | 0.006 |
| Total | -3.37 | <0.001 |

*Note.* Bootstrapped with 500 resamples.
